# Mitochondrial Sequencing of Missing Persons DNA Casework by Implementing Thermo Fisher’s Precision ID mtDNA Whole Genome Assay

**DOI:** 10.3390/genes11111303

**Published:** 2020-11-04

**Authors:** Daniela Cuenca, Jessica Battaglia, Michelle Halsing, Sandra Sheehan

**Affiliations:** California Department of Justice, Jan Bashinski DNA Laboratory, Richmond, CA 94804, USA; Jessica.Battaglia@doj.ca.gov (J.B.); Michelle.Halsing@doj.ca.gov (M.H.); Sandra.Sheehan@doj.ca.gov (S.S.)

**Keywords:** massively parallel sequencing, next-generation sequencing, mitochondrial DNA, forensics, human identification

## Abstract

The advent of massively parallel sequencing (MPS) in the past decade has opened the doors to mitochondrial whole-genome sequencing. Mitochondrial (mt) DNA is used in forensics due to its high copy number per cell and maternal mode of inheritance. Consequently, we have implemented the Thermo Fisher Precision ID mtDNA Whole Genome panel coupled with the Ion Chef™ and Ion S5™ for MPS analysis in the California Department of Justice, Missing Persons DNA Program. Thirty-one mostly challenging samples (degraded, inhibited, low template, or mixed) were evaluated for this study. The majority of these samples generated single source full or partial genome sequences with MPS, providing information in cases where previously there was none. The quantitative and sensitive nature of MPS analysis was beneficial, but also led to detection of low-level contaminants. In addition, we found Precision ID to be more susceptible to inhibition than our legacy Sanger assay. Overall, the success rate (full single source hypervariable regions I and II (HVI/HVII) for Sanger and control region for MPS result) for these challenging samples increased from 32.3% with Sanger sequencing to 74.2% with the Precision ID assay. Considering the increase in success rate, the simple workflow and the higher discriminating potential of whole genome data, the Precision ID platform is a significant improvement for the CA Department of Justice Missing Persons DNA Program.

## 1. Introduction

Mitochondrial (mt) DNA has several distinctions from autosomal DNA that have proven to be beneficial in forensic analysis. First, being the powerhouse of the cell, there are hundreds to thousands of mitochondria in each cell [1,2]. This makes mitochondrial DNA analysis more sensitive than the analysis of nuclear DNA (which only has two copies per diploid cell) and increases the probability of successful PCR amplification [3]. Second, mitochondrial DNA goes through non-Mendelian maternal inheritance and lacks recombination [4,5]. As a conserved marker, it can be used to establish lineage with close or distant kinship. For these reasons, it is widely used to tackle missing person identifications from both routine and extremely challenging samples. The forensic community has historically limited mtDNA analysis to the relatively small (~700 bases) hypervariable regions I and II (HVI and HVII) of the genome. This limitation has been due, in part, to the practical and technical difficulties of sequencing the entire mtGenome using a Sanger sequencing method. Massively parallel sequencing (MPS) addresses this limitation by simplifying whole-genome sequencing [3,6,7,8]. MPS individually sequences millions of fragments at a time and allows molecular barcoding for multiplexing of samples. Without the technical limitations of Sanger sequencing, all 16,569 mtDNA bases are available for sequencing. Whole mtDNA genome studies have been shown to increase the proportion of unique haplotypes from 65–93% when using only the HVI/HVII regions to 90–100% when using whole-genome sequencing [9,10,11,12]. The added discrimination potential and its high sensitivity make mitochondrial DNA analysis with MPS a powerful tool.

The California Department of Justice (CA DOJ), Bureau of Forensic Services, Missing Persons DNA Program receives submissions from unidentified persons, living or deceased, and reference samples from personal effects and family members. For identifications, the extracted DNA is analyzed using autosomal, Y-chromosome, and/or mitochondrial assays. When the DNA is highly limited and/or degraded, autosomal mini-short tandem repeats (STR) fragment analysis and mitochondrial DNA HVI/HVII sequencing have typically been the best analysis tools. Until recently, the Missing Persons DNA Program has used a Sanger sequencing method that utilized two relatively long (~400 bp) HVI/HVII targets for PCR amplification. With this sequencing approach, in some cases of degraded, low-level DNA, mini-STRs have yielded better results than mitochondrial analysis, despite the high copy number benefit of mtDNA [13]. With the recent implementation of the Thermo Fisher Precision ID MPS system, that utilizes smaller amplification targets, we now have greater potential to generate mitochondrial DNA results for highly compromised samples.

The Precision ID system consists of the Precision ID mtDNA Whole Genome Panel, Ion Chef™, Ion S5™ Semiconductor Sequencer, and Converge™ software [14]. Together, the system offers a complete and automated mitochondrial forensic MPS solution. The panel targets the entire mitochondrial genome in 162 relatively small target sequences (163 bp average size) that are amplified in two multiplexed PCR reactions. The Ion Chef™ is a small robot that automates the sample and sequencing preparation steps preceding the sequencing reaction. Sequencing is performed on the Ion S5™ instrument that utilizes Ion Torrent semiconductor chemistry for detection [15]. Data analysis is then conducted on both the Ion S5™ instrument and the local Converge™ server.

Since validating and implementing the Precision ID system, the Missing Persons DNA Program has seen an increase in sequencing success of evidence samples. Here, we discuss our MPS sequencing results from casework samples utilizing the Precision ID system and compare them to corresponding results from our legacy Sanger sequencing approach.

## 2. Materials and Methods

### 2.1. Sample Information

A set of casework and reference samples from the Missing Persons DNA Program were selected for reanalysis with the Precision ID system. The samples were DNA extracts from either casework human remains (bone, hair, nail, or preserved tissue) or reference buccal extracts from possible family members. Bone, hair shaft, and nail samples were cleaned prior to DNA extraction. DNA from hair, nail, and tissue were extracted by an organic method (phenol/chloroform/isoamyl alcohol) and purified in a column. Bone DNA extraction utilized a demineralization procedure and a column filtration [16]. Finally, buccal samples were extracted using the DNA IQ™ Casework Pro Kit for Maxwell^®^ 16 [17].

The samples chosen for this study had previously been analyzed, with varying degrees of success, using our legacy Sanger sequencing approach and/or outsourced to an external sequencing vendor [13]. Some of the casework samples had previously failed using our legacy Sanger sequencing approach, but had been sequenced successfully when outsourced, likely due to the smaller (~200 bp) PCR targets used by the outsourcing vendor for amplifying degraded samples. Although the quality of the reference samples was not challenging, the particular samples were selected based on their haplotypes since they had previously encumbered Sanger sequencing.

Each sample in the study, along with qPCR quantitation results, amplification volumes for Precision ID analysis, and the legacy results for both autosomal STR fragment analysis and mtDNA sequencing are listed in Table 1. Some cases had items that were sampled for extraction multiple times; these samples are grouped together in the table to reflect that they are part of the same case. DNA quantitation was performed using one or more of the following multiplex qPCR assays: Quantifiler Trio™ [18], an internally developed autosomal quadruplex [19], or an internally developed autosomal/mt duplex [20]. Autosomal STR analyses were performed using one or more of the following commercial kits: AmpFlSTR™ Identifiler™ Plus, GlobalFiler™, and/or AmpFlSTR™ MiniFiler [21,22,23]. Legacy mitochondrial Sanger sequencing was performed using Roche’s duplex amplification assay [13]. A subset of the samples in the study were outsourced to a vendor laboratory for HVI/HVII mtDNA analysis. The vendor laboratory used internally developed Sanger sequencing primers, however, the Sanger sequencing approach itself was very similar to the method used by our laboratory.

### 2.2. MPS Library Preparation

The preparation of libraries from DNA extracts was conducted on the Ion Chef™ Instrument following the manufacturer’s protocol [14]. The DNA input used in library preparation was based on several factors that inform the analyst’s decision including an ideal target of 80–100 pg (per our internal validation and manufacturer’s recommendation), the available extract volume, degradation observed in quantitation and STR analysis, inhibition observed at quantitation or STR/mtDNA results and/or extract volume used during Sanger sequencing. Ideal DNA input used for challenging samples has been an ongoing learning process that has evolved during implementation. The volumes used for each sample are listed in Table 1.

The Precision ID mtDNA Whole Genome Panel uses targeted PCR to amplify the whole mitochondrial genome in two large multiplexes. The Ion Chef™ has three library preparation PCR parameters that can be modified: the primer pair number, the number of cycles, and the anneal/extension times. The parameters used for this study were 2 primer pairs, 22 cycles, and 4 anneal and extension times.

For each sample, the products of the two amplification reactions were then pooled for further processing. These products then went through enzymatic reactions to create the libraries for sequencing. Specifically, the amplicons were first partially digested and repaired to make blunt ends. Then the amplicons were A-tailed via an enzymatic non-template base addition to allow for sample-specific adaptor sequences to be added, after which a ligation step was performed. These adaptors contain proprietary sequences that allow for clonal amplification and sequencing, as well as unique barcode sequences for downstream sample identification. Finally, the samples were purified and normalized, then pooled together to create the final library. All of these steps were automated on the Ion Chef™ instrument. In practice, the hands-on steps in library preparation consisted of loading the samples onto a PCR plate, transferring the panel of PCR primers onto a cartridge, and then loading the said plate and cartridge, as well as some other consumables and reagents, onto the Ion Chef™ Instrument. Approximately seven hours later, the library was ready for the next MPS processing step. Each library preparation on the Ion Chef™ is limited to eight samples per automation run. The 31 samples in this study were processed in a total of 14 different Ion Chef™ runs, where each run also contained a positive control (HL60 or 9947A DNA) and a negative control (molecular grade water).

### 2.3. Library Quantitation and Dilution

The concentration of the pooled library that is clonally amplified and subsequently sequenced is crucial for obtaining optimal results. For the types of samples used in this study, i.e., degraded and/or with limited DNA amounts, the initial DNA input for library preparation was not necessarily optimal, which can lead to higher variability in the concentration of the pooled library from the Ion Chef™. For this study, prior to clonal amplification, the pooled libraries were quantified using the Ion Library TaqMan Assay following manufacturer’s guidelines (ThermoFisher, South San Francisco, CA, USA), after which the libraries were diluted to 50 pM [24]. This concentration is higher than the recommended 30 pM concentration for clonal amplification, but was chosen based on internal validation studies that showed that the higher input concentration resulted in a greater number of usable reads (internal validation unpublished). Sequencing-run multiplexes varied from 8 samples (one Ion Chef™ library) to 16 samples (two Ion Chef™ libraries pooled together). The 16 sample runs contained two positive and two negative controls, one per library preparation run.

### 2.4. Emulsion PCR, Ion Chip Loading, and Sequencing

Following quantitation and dilution, the pooled libraries were clonally amplified by emulsion PCR and loaded onto an Ion S5™ 530 sequencing chip. These steps were automated on the Ion Chef™ following the manufacturer’s guidelines [14].

The Ion S5™ instrument was used to sequence the loaded chips. Manufacturer’s recommendations were followed, with the sequencing read length set to 200 base pairs and the flow cycles to 500 [14].

### 2.5. Data Analysis

When sequencing is completed, the Ion S5™ instrument performs some read quality trimming and filtering before bases are called and written to a BAM (Binary Alignment Map) file. These BAM files were analyzed using the mitochondrial module of Converge™ v2.1 software. Converge™ was configured by the vendor to align Precision ID mitochondrial reads and call variants following forensic nomenclature [25]. In addition, Converge™ v2.1 has the capability to report haplogroup estimations. The analysis parameters used for this study varied depending on the number of samples multiplexed. A list of the parameters used in each type of multiplex run is provided in Table 2. These parameters were determined based on internal validation studies (unpublished).

Variant calls are flagged by the software if they do not pass a threshold in Table 2 and/or if they have strand/variant bias. Each sample with flagged variants was evaluated by the analyst. Variant frequency, forward and reverse read and variant balance, haplogroup designation, variant expectancy in haplogroup, and read alignment were all used to evaluate a flagged call. A sample was considered a mixture if two or more read populations were observed in variant-containing portions of the genome, resulting in mixed base calls. One or two mixed bases were accepted as point heteroplasmy if the rest of the genome was consistent with a single source sample.

## 3. Results

The 31 samples were sequenced in a total of 12 runs. Each run varied in the total number of usable reads with a minimum of 7,992,750 and a maximum of 14,961,333 (Table 3).

**Figure 1:**
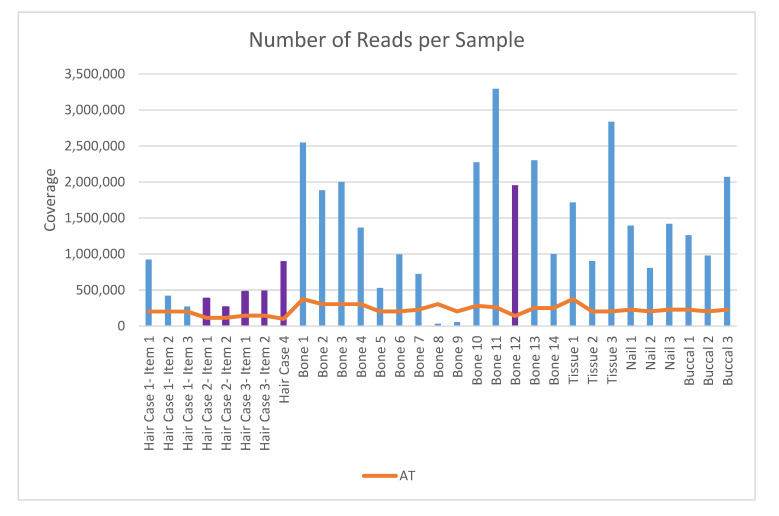
Number of reads per sample and its relation to the analytical threshold (AT). The AT is dynamic in this chart because the samples were not all sequenced in the same run. The AT was determined by multiplying the total usable reads for each run by 0.025 or 0.0125, depending on the number of samples multiplexed in the same run. The value was then plotted on the chart as the orange threshold line. Blue and purple bar distinctions represent samples sequenced in an 8 (blue) or 16 (purple) multiplex.

Based on the number of samples multiplexed together, a run-specific analytical threshold (AT) was applied to each sample in this study. Specifically, for each run, a sample’s percentage of the total usable reads in the run was determined, and analytical thresholds of 2.5% for 8-sample runs and 1.25% for 16-sample runs were applied. This method of calculating each sample’s AT was determined during the internal validation study (unpublished). If a sample did not have enough usable reads to exceed the AT, then it was omitted from further analysis. Figure 1 shows the number of reads per sample and the corresponding analytical thresholds, where the sample nomenclature is the same as presented in Table 1. Bones 8 and 9 fell below the analytical threshold and were not analyzed further. As indicated in Table 1, the extracts for both of these samples had extremely low or undetected quantitation results. Although the Precision ID maximum volume is 15 µL, we opted to use a 10 µL input to be consistent with the legacy procedure, which had a maximum volume of 10 µL.

### 3.1. Hair Results

The success of STR/nuclear DNA analysis in hair is generally dependent on the presence of a hair root and/or adhering tissue [26]. For analysis in the CA DOJ Missing Persons DNA Program, it is general practice to save the hair root, if present, for nuclear DNA testing and to proceed with mtDNA analysis using DNA extract from the shaft. As noted earlier, prior to implementing MPS analysis, our Sanger sequencing approach used a Roche mtDNA Duplex amplification assay that targeted two ~400 bp fragments of HVI and HVII. However, as hair grows, the cells leaving the root will go through DNA degradation/apoptosis [26], sometimes causing DNA testing failure with this assay. Another limitation of hair is the total amount of mtDNA that can be extracted from the shaft. The combination of degraded and limited DNA input makes hairs, in particular hair shafts, challenging to sequence.

Hair samples from four cases (hair cases 1–4, Table 1) were sequenced in this study. In most cases, multiple samplings of the same hair were extracted and sequenced. In case 1, the analyst had sampled three different sections of the same hair shaft. The first extract (item 1) was sampled 2 cm from the proximal root end. The legacy mtDNA Sanger sequencing results for this extract resulted in a mixture. A second sampling (item 2) was excised from the next 2 cm proximal to the root end. The legacy Sanger results for this extract also indicated a mixture, but the mixed base positions of the sequence changed. Due to this unexpected result, a third 2 cm segment was extracted, resulting in yet another sequence mixture, with different mixed-base positions than the first two segments. When these same three extracts were amplified and sequenced with the Precision ID system, the hair segment closest to the root (item 1) produced optimal results, with a full sequence for the whole mtDNA genome (100% of genome had sufficient coverage). This item did show mixed bases at low-levels, but none were called above our 10% threshold, and, therefore, it was considered to be single-source. It may seem unusual that this item had mixed base calls observed in Sanger sequencing (which is less sensitive than MPS). However, these hair samples were limited in input, and it is possible that stochastic effects contributed to these results. The next hair segment (item 2) had 20 mixed-base positions that were called above the mixed base threshold, where 19 of these mixed base calls were C-to-T or G-to-A transitions (18 and 1 calls, respectively) and one was an A-to-G transition observed in two overlapping amplicons at a frequency of 10%. The third hair segment (item 3), most distal from the root, had mixed base calls at 72 positions, 47 of which were C-to-T and 16 of which were G-to-A transitions. None of the mixed-base positions for the three hair segments were the same, and the majority were unique mutations not previously observed in the EMPOP database. It is well known that DNA damage can be seen as deamination that will cause cytosine to resemble uracil, generating a C-to-T or G-to-A replication errors in the PCR [27,28,29,30,31,32,33]. Together, these mixed-base results suggested DNA damage rather than an authentic mixture.

Similarly, the second hair case had resulted in partial and mixed Sanger sequencing results that seemingly resembled DNA damage. The analyst in this case sampled 2 sections of the hair, and the first (item 1) was a 2 cm piece containing the hair root. The second sampling (item 2) was the next 2 cm segment of the shaft. The latter sample went through a locally validated bleach wash to remove potentially exogenous DNA prior to extraction [34]. The hair root sample was not washed in order to preserve any adhering tissue to the root. For MPS, extracts from both of these samples were processed with the Precision ID system. Item 1 had sufficient coverage above threshold for 95% of the mitochondrial genome, including the entire control region. DNA damage could be seen throughout the sample with many C-to-T transitions below the 10% mixed base threshold and two mixed bases that were called just above this threshold. Item 2 resulted in sufficient coverage for 93% of the genome. This item however, had more severe damage with 20 mixed base calls above the 10% threshold, 19 of which were C-to-T or G-to-A changes. None of the mixed base positions for items 1 and 2 were concordant. As in the first hair case, there was an indication of DNA damage in these samples; the farther from the root, the worse the damage. In addition, case 2, item 2 had a low-level third population of reads in known haplotype variant positions that suggested contamination.

Unlike the other hair cases, the third case had no nuclear or mitochondrial legacy results. Three different hairs had been extracted and amplified with the Roche mtDNA Duplex Assay, but no results were obtained. Two new hair samplings (hair case 3, items 1 and 2) from this case were extracted and saved for MPS analysis. Both extracts were processed with the Precision ID mtDNA assay and provided concordant profiles with the exception of a mixed base variant in one hair at 10% frequency. The analyst noted that the mixed base was not a sign of heteroplasmy due to signs of a second contributor at 9–10% frequency. However, the second hair provided a single source sample that was suitable for reporting and Combined DNA Index System (CODIS) upload.

The last hair case in this study (hair case 4) was a hair shaft that had provided full HVI and HVII Sanger sequencing results. When this same hair was processed with MPS, it resulted in a mixed sample in which the major contributor was concordant with the Sanger results. However, due to the contamination and internal policies that prohibit mixture interpretation, the MPS result for this sample was not reported.

The analysis of these challenging hair samples demonstrates the double-edged sword of higher sensitivity. For all of these cases, there were items in which we detected DNA damage or a low-level contaminant that we were unable to detect previously using the Sanger method, leading to unreportable results. Yet, there were also items in all but one case that provided full control region profiles in cases that previously had no reportable STR or mitochondrial results. The evaluation of these hair cases also showed that multiple hair samplings can be beneficial to the successful analysis of the results.

### 3.2. Bones (and Teeth) Results

**Figure 2:**
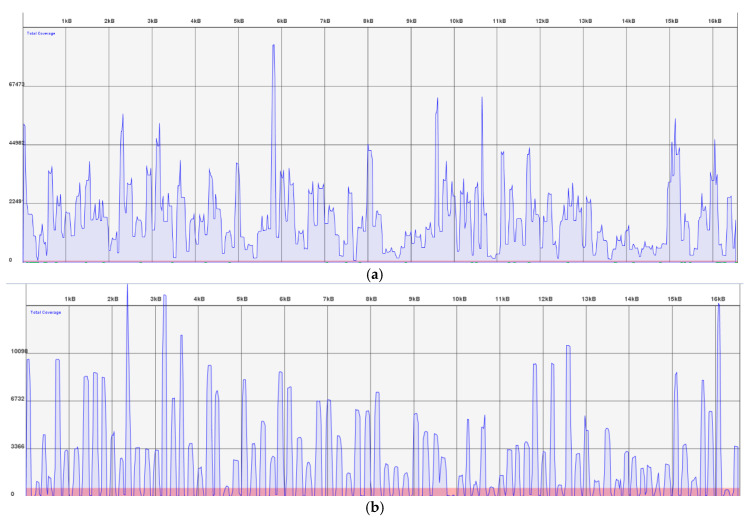
Linear coverage graphs. The x-axis shows the mitochondrial genome from position 1–16,569. The y-axis represents the number of usable reads sequenced. The pink shading shows the coverage threshold of 500 reads. (**a**) Bone 3 amplified well with full coverage for all amplicons. (**b**) Bone 5 is missing coverage for every other amplicon in the panel seen as a disconnected coverage map.

DNA in bones is contained in clusters of minerals that affect extraction recovery. Bones are also often exposed to environmental and/or burial conditions that can cause DNA degradation and inhibition [35,36]. These conditions often make bones a challenging sample set for analysis. In this study, we tested 14 bones with ranging levels of degradation and inhibition. Table 1 shows the results obtained by the legacy STR and mitochondrial sequencing data. In terms of STR results, two bones had full STR profiles (when Minifiler and Identifiler™ or GlobalFiler™ results were combined); the remaining 12 had no to partial STR profiles recovered. For mitochondrial HVI and HVII Sanger sequencing data, the bone samples either had full or partial results, failed completely, or in some cases, were outsourced to target smaller amplicons (~200 bp). When the samples were typed with the Precision ID MPS system, 11 of the 14 bone samples had mitochondrial genome coverage ≥84% (Table 4). For one sample (bone 5), the coverage percentage was 52%. This lack of coverage for approximately half of the genome turned out to be due to a robotic error during Ion Chef™ processing. In particular, the presence of a bubble had prevented automated pooling of the PCR products from one of the two multiplex reactions. Consequently, each alternating amplicon sequence in the genome was missing (Figure 2B). Bone 5 was one of the samples that had not produced any usable data with Sanger sequencing procedures. Since half of the genome was sequenced by MPS despite the robotic error, it is likely that re-processing the extract with the Precision ID could have sequenced much more of the genome. Based on these results, bone 5 will likely be re-sequenced in the future.

**Figure 3:**
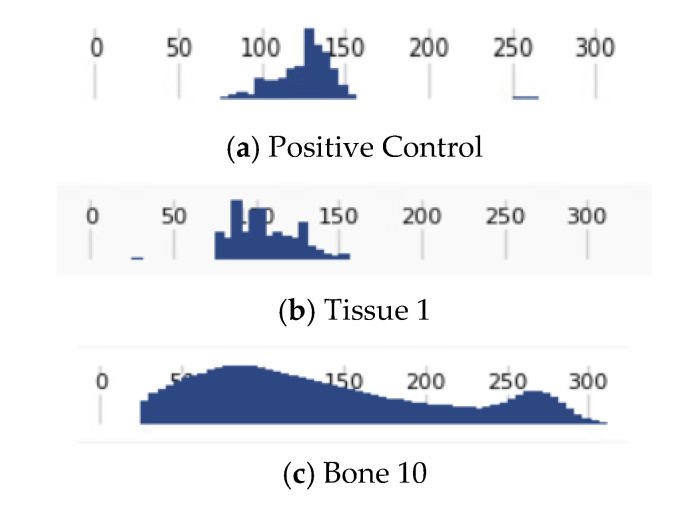
Read histogram from sequencing report. This histogram shows the size of the reads within a sample. (**a**) Histogram for a positive control sample. (**b**) Histogram for tissue 1 showing degradation. (**c**) Histogram for bone sample 10.

A suspected inhibition effect on Precision ID sequencing was seen for two bone samples (bones 10 and 11). Although these samples had more than a million sequencing reads, the percentage of reads that actually aligned to the mitochondrial genome was <1%. Figure 3C shows the amplicon-size histogram report for bone 10 compared to histogram reports for two additional samples from the same sequencing run. These histograms provide a possible explanation for the origin of the low percentage of aligned bone sample reads. For the positive control sample (Figure 3A), the observed histogram distribution spanned an amplicon range of ~75–150 bps, an expected range based on the known target-sequence sizes in the two Precision ID PCR multiplexes. For a tissue sample (tissue 1) in the same sequencing run (Figure 3B), the amplicon size distribution was similar, but skewed toward a relatively lower molecular weight, which is not unexpected, considering that this sample was known to be somewhat degraded. For bone 10 (Figure 3C), however, the observed amplicon size distribution ranged from <50 to 300 bp, which is inconsistent with the expected Precision ID amplicon range and suggestive of non-specific amplification products, possibly of microbial origin. Suspecting PCR inhibition, due to previous Sanger sequencing success, the analyst re-processed the samples targeting a fraction of the initial amplification input to dilute out the inhibitor (66% for bone 10 and 33% for bone 11), which resulted in full mitochondrial genome results. We hypothesize that the presence of inhibitors in the initial bone sample PCR resulted in non-specific amplification of non-mitochondrial DNA, leading to a high number of MPS reads but a very low-level of alignment to the mitochondrial genome. Validation studies of MPS systems have shown that the polymerases used are less robust to inhibition than those typically found in other forensic methodologies [37,38]. Histogram reports may be useful in diagnosing the presence of this type of PCR inhibition in some samples.

### 3.3. FFPE Tissue Results

**Figure 4:**
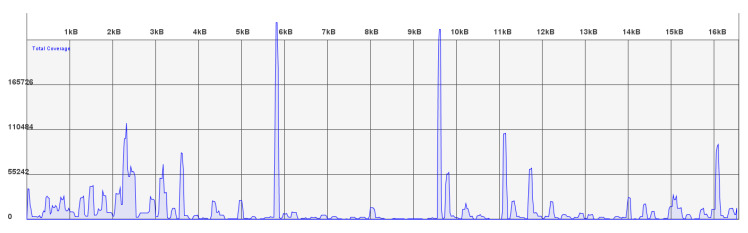
Read coverage for tissue 1. The x-axis shows the mitochondrial genome from position 1–16,569. The y-axis represents the number or reads sequenced. The smallest amplicons in the panel are represented in the coverage map by the two spikes just below 6 and 10 kb.

The Missing Persons DNA Program, on occasion, receives formalin fixed paraffin-embedded (FFPE) tissue. Pathologists use FFPE to preserve tissue. However, the same mechanism that preserves the tissue’s protein structure will also degrade DNA and affect extraction and PCR success [39]. The level of DNA degradation in FFPE samples can vary [40]. The quantitation and legacy results for the tissue samples in this study suggested variable but substantial levels of degradation (Table 1). Yet, the MPS coverages for tissue 1, tissue 2, and tissue 3 were 91.3%, 100%, and 100%, respectively. The read distribution throughout the genome was highly uneven for these samples, most likely due to their high level of degradation. Figure 3 shows the amplicon histograms for an intact sample (positive control) and for tissue 1. The amplicon distribution for tissue 1 is shifted and shows a maximum concentration around 100 bp, compared to ~140 bp for the positive control. Figure 4 shows the coverage map for tissue 1. There are two extremely high coverage areas in the middle of the genome, areas that represent the shortest PCR target sequences in the Precision ID assay. Although a full genome profile was not achievable with MPS for tissue 1, previous Sanger sequencing technology produced no data for this sample. Thus, obtaining sufficient coverage for 91.3% of the mtDNA genome, including the full control region, was a significant improvement.

### 3.4. Nail and Buccal Swab Results

**Figure 5:**
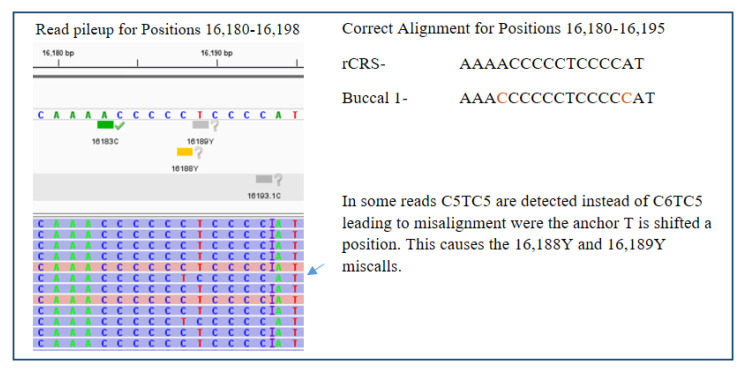
Precision ID homopolymer detection causing an alignment miscall at positions 16,188 and 16189. The insertion at position 16,193 is represented as a purple i.

Nail and buccal swab samples are common tissue types tested in the Missing Persons DNA Program. For this study, samples were selected that either had haplotype sequences that were difficult to interpret with Sanger sequencing, i.e., long or unusual homopolymer stretches or had degraded DNA. Long homopolymer stretches continue to be a challenge to sequence. When performing legacy Sanger technology, it was typically necessary to utilize additional primers to target the area surrounding the homopolymer. With semiconductor detection technology, as used by the Ion S5™ sequencers, homopolymer stretches are read in the same base flow, and the number of bases is determined by the intensity of the signal. However, for long homopolymer stretches, the error rate for this sequence motif is high, leading to ambiguity in the number of bases detected [41]. The haplotypes for nail 1 and buccal 2 have long HVI C-stretch sequences in which we observed ambiguity in the number of bases detected by MPS. Per our internal protocol and SWGDAM recommendations, the number of cytosine bases in the HVI C-stretch is not determined [42]. Nonetheless, alignment and variant calling in this section should follow phylogenetic rules. Buccal 1 had alignment issues that caused miscalls due to an insertion at position 16,189.1C. Figure 5 shows this section and the misalignment causing miscalls at positions 16,188 and 16,189. Buccal 3 had an HVII haplotype that results in a stretch of 13 cytosine bases. Only 50% of the reads in buccal 3 detected the 13 C stretch. The rest of the reads detected 12 and 11 cytosine bases. Nail 3 was suspected of having degradation or very low-levels of input, as it had yielded low quantitation and no legacy results. When it was sequenced with the Precision ID system, it provided sufficient coverage for the whole mitochondrial genome.

These results confirmed what had been observed in the internal validation of the Precision ID system. Homopolymer stretches are challenging for this technology and, in some cases, can lead to misalignment and variant miscalls. Analysts will have to take caution in these areas to correct miscalled variants in accordance to phylogenetic and SWGDAM recommendations. Fortunately, none of these miscalls would have resulted in a false exclusion during a comparison given the nature and location of the variants.

A summary of the results obtained for each sample in the study is listed in Table 4. The table shows the percent genome coverage obtained, CA DOJ Sanger sequencing success, CA DOJ plus outsourcing Sanger sequencing success, and Precision ID MPS sequencing success.

## 4. Discussion

This study is an evaluation of the sequencing success rate for the newly implemented Precision ID mtDNA Whole Genome Assay and a comparison against legacy Sanger sequencing methodology. The case samples selected were intentionally challenging and most had low success rates with the Sanger sequencing method. For the purpose of this discussion, successful sequencing is defined as a full single-source mitochondrial HVI/HVII haplotype for Sanger sequencing and a full single-source control region (CR) haplotype for MPS results. Although we collected sequence data for the full mitochondrial genome with the Precision ID assay, the control region is the only portion that is currently uploaded into CODIS and is, therefore, most relevant when comparing relative success rates.

The Precision ID and Sanger sequencing success rates for the set of samples included in this study are summarized in Table 4. The coverage for the whole genome is also included in the table to serve as an indication of the quality of the sample/results. The CA DOJ success rate with legacy Sanger sequencing for the cases presented here was 25% (7/28). As previously mentioned, some of the samples in this study were outsourced to a laboratory that utilized shorter amplification targets. Therefore, some of the samples that had failed at CA DOJ were successfully sequenced when outsourced. The success rate for CA DOJ including the samples that succeeded when outsourced was 32.3% (10/31). The implementation of Precision ID mtDNA increased the success rate to 74.2% (23/31). All but one sample that failed Precision ID mtDNA sequencing had also failed Sanger sequencing (at CA DOJ and/or the outsourcing lab) and were either highly damaged hair samples or bone samples that had very low quantitation results. Some of these cases had potential PCR inhibition that failed amplification on the first attempt. Sample (and inhibitor) dilutions were able to improve the results. Inhibition should be kept in mind when using this technology as the assay may be unable to overcome such conditions. Moving forward, the incorporation of extra cleaning procedures (e.g., NucleoSpin columns) may be useful when inhibition is observed at quantitation and/or during STR or mtDNA amplification. Similarly, MPS was able to improve the success rate with hair shaft samples but it is unable to overcome the high level of damage that can occur. A high number of C-to-T and G-to-A nucleotide transitions was observed in these samples. DNA damage can be identified by these preferential base changes and their random placement throughout the genome. In addition, many of these transitions are in positions that have never been observed in the EMPOP database. A DNA repair procedure could improve these results [27] and therefore allow us to more easily interpret them. However, CA DOJ does not currently have any DNA repair methodology validated.

The one sample that provided better results with Sanger sequencing over MPS was a hair shaft that had mixed DNA results. For this sample, it is possible that DNA contamination was introduced during the MPS protocol, but it is more likely that the initial extract had a low-level contaminant that was below the detection levels of the less sensitive Sanger sequencing method.

In summary, CA DOJ implementation of the Precision ID MPS workflow has led to two significant improvements for mtDNA sequencing analysis. In contrast to our legacy Sanger sequencing workflow, which was highly manual and labor intensive, nearly all of the pre-sequencing Precision ID chemistry is automated on the Ion Chef™, significantly saving laboratory time and labor for the analysts, as well as reducing the time required for training and implementation. This has allowed the Missing Persons DNA Program to quickly work their way through priority samples, allowing them to start working on older cases that had been backlogged. In addition, the use of the Precision ID MPS workflow led to a significant increase in our capability to successfully sequence challenging samples, particularly for degraded samples of the type that are often encountered in the Missing Persons DNA Program.

## Figures and Tables

**Table 1:** Samples Description Table. The table shows sample name, sample type, DNA concentration, legacy results, and Precision ID (PID) amplification volume used. STR and Sanger sequencing was attempted for all samples, “No STR” or “No HVI/HVII results” indicate unsuccessful results. Not every sample was quantified with both autosomal and mitochondrial targets; cells that show N/A indicate that sample was not quantified using that target. In the Volume Used column dilution is abbreviated to Dil.

		qPCR Sample Quantitation				
Sample Name	Sample Type	Large Target (pg/µL)	Small Target (pg/µL)	mt Target (mtCopies/µL)	Legacy Results (Nuclear and Mitochondrial)	Precision ID Volume Used (µL)
Hair Case 1—Item 1	Hair—2 cm proximal end	0	Not Applicable (N/A)	16,000	No short tandem repeat (STR); hypervariable regions I/II (HVI/HVII) Mixture	3.4 ^1^
Hair Case 1—Item 2	Hair Shaft—2 cm after item 1 sampling	0	N/A	2700	No STR; HVI/HVII Mixture	2.8 ^1^
Hair Case 1—Item 3	Hair Shaft—2 cm after item 2 sampling	0	N/A	160	No STR; HVI/HVII Mixture	3.3 ^1^
Hair Case 2—Item 1	Hair Root + Shaft	0	0	N/A	No STR; Partial HVI/HVII Results and a Mixture	15 ^1^
Hair Case 2—Item 2	Hair Shaft—2 cm after root	0	0	N/A	No STR; Partial HVI/HVII Results and a Mixture	15 ^1^
Hair Case 3—Item 1	Hair Shaft	N/A	N/A	N/A	No STR; No HVI/HVII Results	15 ^1^
Hair Case 3—Item 2	2nd Hair Shaft	N/A	N/A	N/A	No STR; No HVI/HVII Results	15 ^1^
Hair Case 4	Hair Shaft	N/A	N/A	3525	HVI and HVII Results	15 ^1^
Bone 1	Tooth	22.1	49.3	13,650	Full STR Identifiler + MiniFiler; HVI/HVII Results Outsourced	10 ^2^
Bone 2	Foot Bone	0.1	0.5	1935	Full STR Identifiler + MiniFiler; HVI/HVII Results Outsourced	6.5 ^1^
Bone 3	Femur	0.5	1.1	6550	No STR; HVI/HVII Results Outsourced	6.5 ^1^
Bone 4	Skull	0.1	0.2	670	No STR; Partial HVI/HVII Results	8 ^1^
Bone 5	Femur	0	0	N/A	No STR; No HVI/HVII Results	7.2 ^1^
Bone 6	Femur	0	N/A	1890	3 STRs Identifiler + MiniFiler; No HVI/HVII Results	13.2 ^1^
Bone 7	Pelvic Bone	0	7200	N/A	No STR; No HVI/HVII Results	10 ^2^
Bone 8	Tooth	0	0	3.34	No STR; No HVI/HVII Results	10 ^2^
Bone 9	Tooth	0	48	N/A	No STR; HVI/HVII Results Outsourced	10 ^2^
Bone 10	Femur	2.5	5.3	N/A	Partial STR; HVI/HVII Results	15 ^1^
Bone 11	Femur	71	30,000	N/A	Partial STR; No HVI/HVII Results	5 ^4^
Bone 12	Tooth	4.6	71.4	N/A	Partial STR; No HVI/HVII Results	5 ^4^
Bone 13	Tooth	0	170	N/A	Partial STR; No HVI/HVII Results	3 ^4^
Bone 14	Femur	88.1	132	28,000	Partial STR; No HVI/HVII Results	1 ^3^
Tissue 1	Tissue	0	231	895,000	No STR; Partial mtDNA Results	10 ^2^
Tissue 2	Tissue	0.1	658	N/A	Partial MiniFiler; No HVI/HVII Results	1 ^4^
Tissue 3	Tissue	N/A	N/A	N/A	Full STR with MiniFiler; Partial HVI/HVII Results	15 ^1^
Nail 1	Nail	370	N/A	17,000,000	Full STR; HVI/HVII Results	1 of 1/3 Dil ^3^
Nail 2	Nail	8900	53,000	N/A	Full STR; HVI/HVII Results	1 of 1/90 Dil ^3^
Nail 3	Nail	0	41	N/A	No STR; No HVI/HVII Results	8.5 ^1^
Buccal 1	Buccal	180	260	N/A	Full STR; HVI/HVII Results	1 of 1/2 Dil ^3^
Buccal 2	Buccal	460	560	N/A	Full STR; HVI/HVII Results	1 of 1/4 Dil ^3^
Buccal 3	Buccal	320	360	N/A	Full STR; HVI/HVII Results	1 of 1/3 Dil ^3^

^1^ Maximum available DNA extract volume used. ^2^ Volume used equals what was used for Sanger sequencing. ^3^ A target of 80–100 picograms was used. ^4^ Volume used based on quantitation and degradation index (concentration of small DNA target/concentration of large DNA target) results.

**Table 2:** Converge™ v2.1 analysis settings for sequencing runs of 8 and 16 samples. For the minimum coverage percent, the threshold is relative to the median number of reads for the amplicon of interest. The confirmed, point heteroplasmy, insertion and deletion percentage thresholds are related to the total number of reads for the base position of interest.

Converge v2.1 Analysis Setting	8 Sample Multiplex	**16 Sample Multiplex**
Minimum Total Read Coverage per Base Position	500 reads	250 reads
Minimum Variants Coverage to Call	50 reads	25 reads
Coverage Threshold to Mark Region	500 reads	250 reads
Minimum Coverage Percent	5%	5%
Confirmed Call Threshold	90%	90%
Point Heteroplasmy Call or Mixed Base Threshold Call Threshold	10%	10%
Insertion Call Threshold	20%	20%
Deletion Call Threshold	30%	30%

**Table 3:** Sequencing-run metrics. Chip loading refers to the percent of wells in the chip that have a DNA containing bead. Polyclonal percentage is the percent of wells that have a mixed signal or had two or more DNA fragments during clonal amplification. Percent of low-quality reads refers to the percentage of reads filtered out by quality filters. The last metric is the total number of usable reads after all the filters are applied.

Run Number	Chip Loading (%)	% Polyclonal	% Low Quality Reads	Usable Reads
Run 1	77	38	26	12,174,997
Run 2	87	42	19	14,961,333
Run 3	84	37	52	8,050,849
Run 4	90	43	57	8,135,888
Run 5	84	45	47	9,107,278
Run 6	83	47	28	11,581,237
Run 7	87	54	33	9,079,330
Run 8	83	55	33	7,992,750
Run 9	88	51	27	11,247,818
Run 10	89	46	38	10,473,906
Run 11	89	49	27	11,248,640
Run 12	91	55	29	10,002,927

**Table 4:** Precision ID mtDNA assay success rate. The table shows the sample name, Precision ID result coverage percentage for the full genome, California Department of Justice (CA DOJ) Sanger results, CA DOJ and outsourcing combined Sanger results, the Precision ID assay results and the success rate for each. Success rate is determined based on a full single-source HVI and HVII Sanger result or a full control region massively parallel sequencing (MPS) result. The absence of a box indicates no typing attempt was made, the presence of an uncrossed box (☐) indicates typing was attempted but unsuccessful, and a crossed box (☒) indicates typing was attempted and successful.

Sample Name	Full mtDNA Coverage	Sanger CA DOJ	Sanger CA DOJ and Outsourcing Combined Results	Precision ID Assay
Hair 1–1	100%	☐	☐	☒
Hair 1–2	93%	☐	☐	☐
Hair 1–3	75%	☐	☐	☐
Hair 2–1	95%	☐	☐	☒
Hair 2–2	93%	☐	☐	☐
Hair 3–1	96%	☐	☐	☐
Hair 3–2	93%	☐	☐	☒
Hair 4	99.4%	☒	☒	☐
Bone 1	99.5%		☒	☒
Bone 2	99.4%		☒	☒
Bone 3	100%	☐	☒	☒
Bone 4	98.8%	☐	☐	☒
Bone 5	52% *	☐	☐	☐ *
Bone 6	96.7%	☐	☐	☒
Bone 7	84%	☐	☐	☒
Bone 8	0%	☐	☐	☐
Bone 9	0%		☐	☐
Bone 10	85%	☒	☒	☒
Bone 11	100%	☐	☐	☒
Bone 12	100%	☐	☐	☒
Bone 13	99.5%	☐	☐	☒
Bone 14	100%	☐	☐	☒
Tissue 1	91.3%	☐	☐	☒
Tissue 2	100%	☐	☐	☒
Tissue 3	100%	☐	☐	☒
Nail 1	100%	☒	☒	☒
Nail 2	100%	☒	☒	☒
Nail 3	100%	☐	☐	☒
Buccal 1	99.2%	☒	☒	☒
Buccal 2	100%	☒	☒	☒
Buccal 3	85%	☒	☒	☒
Success	Rate	7/28 = 25%	10/31 = 32.3%	23/31 = 74.2%

* Bone 5—sample affected by the robotic error. It is marked unsuccessful for MPS results because it did not have a full control region. However, as discussed in Section 3.2, this is likely due to an automation error and not the sample.
